# Acute Adverse Effects of Therapeutic Doses of Psilocybin: A Systematic Review and Meta-Analysis

**DOI:** 10.1001/jamanetworkopen.2024.5960

**Published:** 2024-04-10

**Authors:** Akhila Yerubandi, Jennifer E. Thomas, N. M. Mahmudul Alam Bhuiya, Catherine Harrington, Lorenzo Villa Zapata, Joshua Caballero

**Affiliations:** 1 Department of Clinical and Administrative Pharmacy, College of Pharmacy, University of Georgia, Athens; 2 Department of Clinical and Administrative Sciences, Larkin University, Miami, Florida; 3 Lloyd L. Gregory School of Pharmacy, Palm Beach Atlantic University, West Palm Beach, Florida

## Abstract

**Importance:**

Psilocybin has been studied in the treatment of depression and anxiety disorders. Clinical studies have mainly focused on efficacy, with systematic reviews showing favorable efficacy; however, none have primarily focused on psilocybin safety.

**Objective:**

To evaluate the acute adverse effects of psilocybin at therapeutic doses in the treatment of depression and anxiety.

**Data Sources:**

MEDLINE via PubMed, Web of Science, and ClinicalTrials.gov were searched for publications available between 1966 and November 30, 2023.

**Study Selection:**

Randomized, double-blind clinical trials that reported adverse effects of psilocybin in patients treated for depression and anxiety were screened.

**Data Extraction and Synthesis:**

Data were independently extracted by 2 authors and verified by 2 additional authors following the Preferred Reporting Items for Systematic Reviews and Meta-Analyses guideline. The inverse variance method with the Hartung-Knapp adjustment for the random-effects model was used, with a continuity correction of 0.5 for studies with 0 cell frequencies. Sensitivity analysis was conducted by sequentially removing 1 study at a time to assess the robustness of the results.

**Main Outcomes and Measures:**

The primary outcome was considered as the adverse effects of psilocybin at high and moderate (ie, therapeutic) dose regimens and compared with placebo, low-dose psilocybin, or other comparator in the treatment of depression and/or anxiety.

**Results:**

Six studies met the inclusion criteria with a total sample of 528 participants (approximately 51% female; median age 39.8 years; IQR, 39.8-41.2). Seven adverse effects were reported in multiple studies and included in the analysis. Among these, headache (relative risk [RR], 1.99; 95% CI 1.06-3.74), nausea (RR, 8.85; 95% CI, 5.68-13.79), anxiety (RR, 2.27; 95% CI, 1.11-4.64), dizziness (RR, 5.81; 95% CI, 1.02-33.03), and elevated blood pressure (RR, 2.29; 95% CI, 1.15- 4.53) were statistically significant. Psilocybin use was not associated with risk of paranoia and transient thought disorder.

**Conclusions and Relevance:**

In this meta-analysis, the acute adverse effect profile of therapeutic single-dose psilocybin appeared to be tolerable and resolved within 48 hours. However, future studies need to more actively evaluate the appropriate management of adverse effects.

## Key Points

**Question:**

What are the notable acute adverse effects for therapeutic doses of psilocybin in the treatment of depression and anxiety?

**Findings:**

In this meta-analysis of 6 randomized, double-blind clinical trials with 528 patients, headaches, nausea, anxiety, dizziness, and fluctuations in blood pressure occurred significantly more frequently with psilocybin vs comparators. Psilocybin use was not associated with risk of paranoia and transient thought disorder.

**Meaning:**

The findings of this study suggest a tolerable acute adverse effect profile for therapeutic doses of psilocybin, but rare and long-term adverse effects need to be further elucidated.

## Teaser

This systematic review and meta-analysis examines the incidence of acute adverse effects observed with the administration of psilocybin in the treatment of depression and anxiety.

## Introduction

Psilocybin is classified as a serotonergic psychedelic and a prodrug of psilocin (4-hydroxy-dimethyltryptamine), which converts to the active form once ingested.^1^ The theoretical mechanism of action involves binding to serotonin_2A_ (5-HT_2a_) predominantly in the amygdala, thalamus, and prefrontal cortex. The psychopharmacologic profile of psilocybin was examined in the 1960s. It was proposed that oral administration of approximately 10 mg was needed to induce psychological effects, with more potent effects developing with increasing doses.^2,3,4^ Psilocybin’s psychological effects were comparable to those of lysergic acid diethylamide, but thought to be more vividly visual, less emotionally intense, more euphoric, and less likely to cause panic attacks or paranoia.^4^ Clinical studies suggest psilocybin produces an antidepressant benefit in patients with treatment-resistant depression.^5^ This impact is believed to be connected to its affinity for the serotonergic pathway in the brain, which is essential in controlling mood.^5^

In recent years, there has been renewed interest in the therapeutic potential of psilocybin in the treatment of mental health (eg, depression, anxiety) disorders.^6,7,8^ Psilocybin-assisted therapy typically involves 1 or 2 dosing sessions with individuals encouraged to explore their thoughts and emotions with the support of a therapist. Clinical studies have focused on psilocybin efficacy, resulting in studies pooling and presenting aggregate results.^6,7^ One recent meta-analysis investigated psilocybin adverse effects as a secondary aim, using a dose-dependent approach focused on select adverse effects.^8^ However, these studies have not primarily focused on or explored the adverse effect profile of psilocybin in depth.^6,7,8^ Therefore, the purpose of this study was to summarize and examine the relative risk (RR) of acute adverse effects of therapeutic doses of psilocybin in patients with depression and anxiety.

## Methods

A systematic review of the literature following the Preferred Reporting Items for Systematic Reviews and Meta-Analyses (PRISMA) reporting guideline^9^ was conducted to identify studies involving participants receiving psilocybin in the treatment of major depressive disorder or depression associated with other related disorders (eg, cancer-related anxiety and depression). Studies included randomized clinical trials comparing psilocybin with either placebo or another comparator (eg, niacin, escitalopram, low-dose psilocybin). Doses were grouped into low (1-3 mg), moderate (10-20 mg), and high (20-30 mg) categories. These dosing ranges were based on previous clinical data.^10,11^ All studies were evaluated for adverse effects of psilocybin in the treatment of depression and anxiety in study participants. When selecting adverse event profile rates, the shortest time period available was selected and analyzed (eg, day 1 instead of day 30) since the half-life of psilocin is 3 ± 1.1 hours when taken orally and the duration of action can range between 3 to 12 hours.^12,13^ Therefore, it is expected that psilocin concentrations would be minimal by 24 hours.

Published studies were identified by conducting a search of MEDLINE via PubMed, Web of Science, and ClinicalTrials.gov for publications available between 1966 and November 30, 2023. Search terms included *psilocybin*, *side effects*, *depression*, *anxiety*, *adverse effects*, and *adverse side effects*. Only randomized, double-blind clinical trials published in the English language were included in the systematic review. Studies were included in the analysis if they reported the adverse effects of psilocybin. Studies were excluded if they reported the same adverse events from previous studies or follow-up studies in which adverse event data were already described (eg, deferring adverse event profile to most recent exposure). When appropriate and based on our expertise, adverse effects having different terminology but similar definitions were grouped. These included nausea and transient nausea, headache and transient headache, anxiety and transient anxiety, paranoia and paranoid ideation, and transient thought disorder and abnormal thinking.

Data were independently extracted by 2 of us (A.Y. and N.M.M.A.B.) and verified by an additional 2 of us (J.E.T. and J.C.). The primary outcome was considered the adverse effects of psilocybin at high- and moderate-dose regimens (ie, therapeutic doses) and compared with placebo, low-dose psilocybin, or another comparator in the treatment of depression and anxiety with other related disorders.

### Statistical Analysis

The meta-analysis was conducted using R statistical software, version 4.3.1 (R Foundation for Statistical Computing). We used the inverse variance method with the Hartung-Knapp adjustment for the random-effects model and used a continuity correction of 0.5 for studies with 0 cell frequencies. Sensitivity analysis was conducted by sequentially removing 1 study at a time to assess the robustness of our results. For outcomes with 4 or less studies, a common effect model was considered. Heterogeneity among the studies was quantified using the *I*^2^ measure. Additionally, for each study, a funnel plot was created to evaluate publication bias. Study quality was assessed by the risk-of-bias tool for randomized trials (RoB2).^14^ Two of us (A.Y. and N.M.M.A.B) determined initial RoB2 scores for the included studies. An additional 2 of us (J.C. and J.E.T.) then independently assessed and verified initial scores. Any discrepancies were discussed and rescored as needed. With 2-sided analysis, the significance threshold was *P* ≤ .05.

## Results

**Figure 1.**
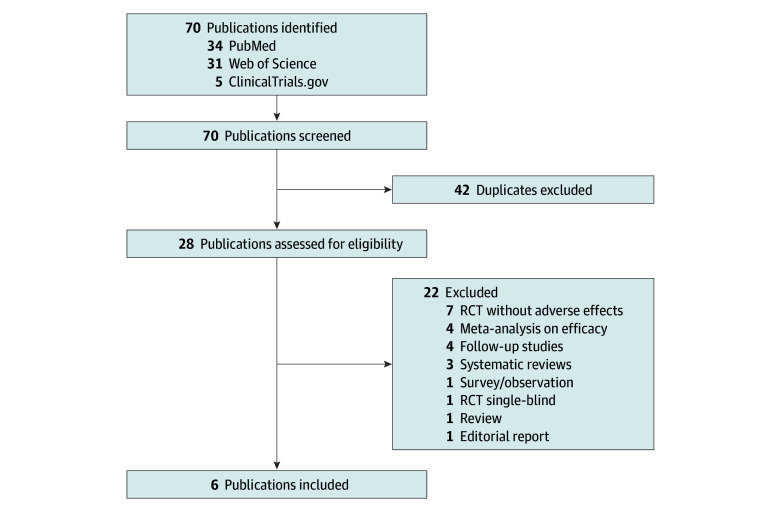
Flow Diagram for New Systematic Reviews That Included Searches of Only Databases and Registers RCT indicates randomized clinical trial.

Overall, of 70 published studies identified through PubMed, Web of Science, and ClinicalTrials.gov, 64 studies were excluded (Figure 1). Therefore, 6 studies (total sample of 528 participants; approximately 51% female; 49% male; median age, 39.8 [IQR, 39.8-41.2] years) met our inclusion criteria (Table).^11,15,16,17,18,19^ In general, the population was middle-aged adults and more than 90% of the participants were White. The RoB2 assessment tool showed an overall low risk of bias for all included studies. Several adverse effects were identified throughout the studies. Comparators identified in these studies included placebo, niacin, escitalopram, and low-dose psilocybin (1-3 mg). In general, participants experienced adverse effects immediately or within 24 hours after administration of various doses of psilocybin.

**Table.** Summary of Included Studies

Source	No. of participants	Mean age, y	Gender	Psilocybin dosing	Comparator	Disease state	Notable psilocybin adverse effects
Goodwin et al, ^11^ 2022	79 (high dose), 75 (moderate dose), 79 (low dose)	39.8	52% Female	10 mg, 25 mg	Low dose psilocybin (1 mg)	Treatment-resistant depression	Headache with high (24%) vs moderate (15%) vs low (16%) dose, nausea with high (22%) vs moderate (7%) vs low (1%) dose, anxiety with high (4%), moderate (8%), and low (0%) dose; dizziness with high dose (6%), fatigue with high dose (6%), euphoric mood with high (5%), altered mood with high (5%)
Raison et al,^15^ 2023	104	41.1	47% Female	25 mg	Niacin	Major depressive disorder (24% had treatment-resistant depression)	Headache (66%), nausea (48%), visual perceptual effects (44%)
Griffiths et al,^16^ 2016	51^a^	56.3	49% Female	22 mg	Low dose psilocybin (1 mg)	Depression and/or anxiety in cancer	Elevated systolic BP with high (34%) vs low (17%) dose, elevated diastolic BP with high (13%) vs low (2%) dose, headache with high (2%) vs low (0%) dose, nausea/vomiting with high (15%) vs low (0%) dose, physical discomfort with high (21%) vs low (8%) dose, anxiety with high (26%) vs low (15%) dose
Ross et al,^17^2016	29^b^	56.3	62% Female	21 mg	Niacin	Depression and/or anxiety in cancer	Nonclinically significant elevated BP and heart rate (76%), headache (28%), nausea (14%), transient anxiety (17%), transient psychoticlike symptoms (7%)
Carhart-Harris et al,^18^ 2021	59	41.2	32% Female	25 mg	Escitalopram plus low-dose psilocybin (1 mg)	Major depressive disorder	Headache with high dose (43%) vs low dose plus escitalopram (17%), nausea with high dose (13%) vs low dose plus escitalopram (0%)
von Rotz et al,^19^2022	52	36.8	63.5% Female	0.215 mg/kg body weight (estimated 15-16 mg)	Placebo	Major depressive disorder	Headache (15%), dizziness (8%), nausea (4%)

Abbreviation: BP, blood pressure.

^a^ Study states overall sample size of 51 for completing session 1; however, a sample size of 50 per group was used in meta-analysis per data presented in Figure 1 for data obtained.

^b^ Study states overall sample size of 29 completing; however, a sample size of 28 (experimental) and 27 (control) was used in meta-analysis per data presented in Figure 1 for data obtained.

**Figure 2.**
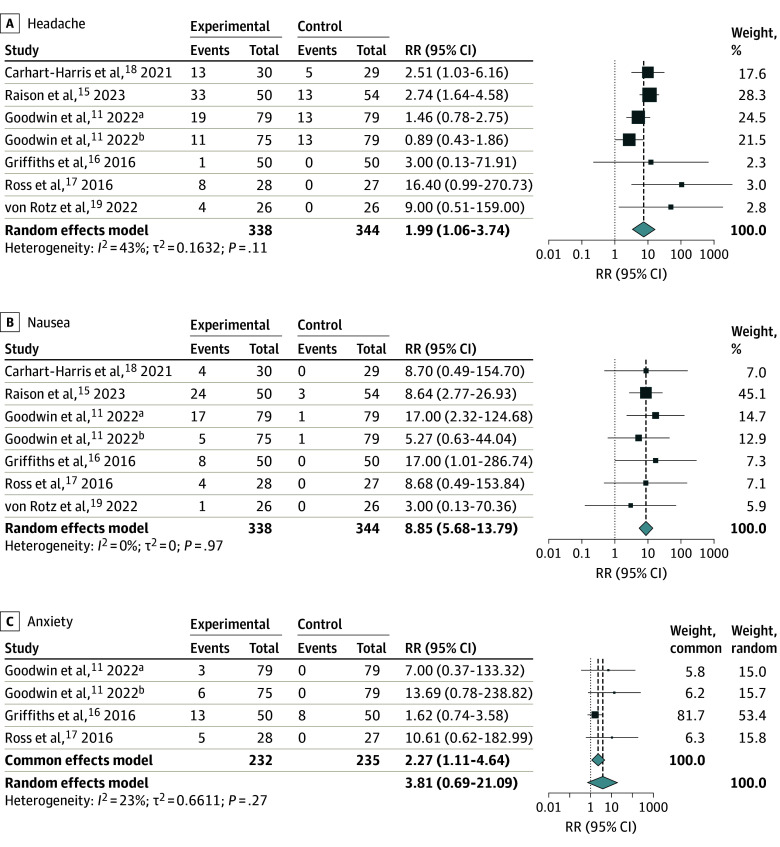
Association of Psilocybin With Headache, Nausea, and Anxiety Bold copy emphasizes the random-effects model and common effects model. Square size indicates the weight of the study; diamonds, the total weight. ADE indicates adverse drug effect; RR, risk ratio. ^a^High-dose psilocybin. ^b^Moderate-dose psilocybin.

**Figure 3.**
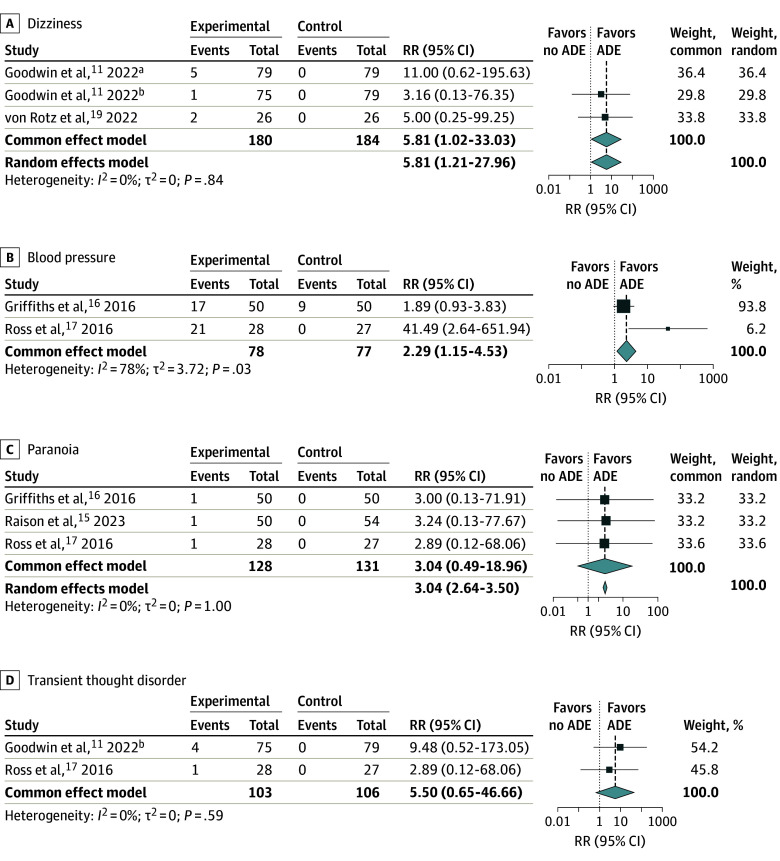
Association of Psilocybin With Dizziness, Blood Pressure, Paranoia, and Transient Thought Disorders Bold copy emphasizes the random-effects model and common effects model. Square size indicates the weight of the study; diamonds, the total weight. ADE indicates adverse drug effect; RR, risk ratio. ^a^High-dose psilocybin. ^b^Moderate-dose psilocybin.

Safety data (Figure 2 and Figure 3) from the randomized clinical trials to assess risk ratio via meta-analysis were available for the following 7 adverse effects: headache, nausea, anxiety, dizziness, paranoia, transient thought disorder, and elevated blood pressure. Overall, psilocybin was associated with a greater risk of adverse effects of headache (RR, 1.99; 95% CI, 1.06-3.74; *P* = .04), nausea (RR, 8.85; 95% CI, 5.68-13.79; *P* < .001), anxiety (RR, 2.27; 95% CI, 1.11-4.64; *P* = .02), dizziness (RR, 5.81; 95% CI, 1.02-33.03; *P* = .047), and elevated blood pressure (RR, 2.29; 95% CI, 1.15-4.53; *P* = .02) compared with control. Psilocybin use was not associated with risk of paranoia and transient thought disorder. Overall, 2 adverse effects appeared in all 6 studies, including headache, with an incidence ranging from 2% to 66% and nausea with an occurrence varying from 4% to 48%.^11,15,16,17,18,19^ Anxiety was documented in 3 studies, with an incidence ranging from 4% to 26%.^11,16,17^ All adverse effects had an estimated *I*^2^ value of less than 50%, except elevated blood pressure (*I*^2^ = 78%), suggesting most results were not affected by heterogeneity. In the sensitivity analysis, minor adjustments in RR were observed for headache and nausea, while anxiety, dizziness, paranoia, and transient thought disorder showed unchanged RR values. This consistency, despite limited studies for some conditions, highlights the robustness of our findings. Sensitivity and funnel plot analysis are located in eFigure 1 and eFigure 2 in Supplement 1.

Additionally, there were 6 acute adverse effects that appeared in greater than 5% of the population, including elevated heart rate (76%), visual perceptual effects (44%), physical discomfort (21%), fatigue (approximately 6%), euphoric mood (approximately 5%), and mood alteration (approximately 5%).^11,15,16,17^ However, these adverse effects were identified in only 1 study and therefore not included in the meta-analysis. The studies reported none of the adverse events listed were considered serious. A summary of the studies is presented in the Table.

## Discussion

A summary of the acute adverse effects of psilocybin in treating depression and anxiety is needed for health care professionals to identify expected adverse effects and provide effective patient counseling. This study focused on therapeutic doses to clarify the expected adverse effects in potential future practice. The results overall suggest a statistically significant incidence of headache, nausea, anxiety, dizziness, and elevated blood pressure. However, caution is advised in interpreting elevated blood pressure due to its heterogeneity (*I*^2^ = 78%), indicating potential variability. Given the psilocybin mechanism of action, these adverse effects are expected as they are similar among serotonergic antidepressants.^1^ The adverse effects were also anticipated based on previous survey data from adult participants who ingested active doses of psilocybin mushrooms.^20^ Additionally, data on adverse effect severity appear to align with documented psychedelic adverse effects over the past 60 years or more.^21,22,23^

### Headache

All 6 studies identified headache as a statistically significant adverse effect of psilocybin, with an incidence ranging from 2% to 66%.^11,15,16,17,18,19^ Headaches were typically mild to moderate in severity, and none required medications for relief. Literature reports corroborate headaches as a known adverse effect of psilocybin. A recent study found a significant dose-response relationship, with the RR of headaches/migraines increasing by 1.42% for each unit increase in psilocybin dose.^8^ Moreover, a small double-blind study in healthy individuals indicated a dose-related response to psilocybin with respect to headache occurrence, duration, and intensity.^24^ These headaches subsided within 24 hours of psilocybin administration.

### Nausea

All 6 studies identified nausea as an adverse effect.^11,15,16,17,18,19^ Incidence varied between 4% and 48%.^15,18^ One study reported nausea at 22% with a high dose, but the rate decreased with lower doses (eg, 7% with moderate dose, 1% with low dose).^11^ An additional study reported 15% of participants experienced nausea at a high dose and none with a low dose.^16^ Recent data support a dose-response association with a relative risk of 1.25% (*P* < .001).^8^ Five studies stated nausea was not severe and resolved within 60 minutes.^11,16,17,18,19^ While the severity of nausea was not discussed in the studies, none reported using any pharmacologic agent to assist with nausea. Additionally, medications to alleviate any severe nausea or vomiting, if needed, were not identified in the study protocols. There are anecdotal reports suggesting eating 1 to 2 hours before taking psilocybin or taking with a small snack, using lemon juice and/or ginger, and hydrating well may be helpful.^25,26^ Others advise patients can concentrate on themselves in an act of surrendering to the psychedelic experience.^27,28^ However, there are no clinical studies to support these suggestions or allopathic treatments and caution is warranted. For example, ginger may potentiate the effects of psilocybin due to its ability to increase serotonin and produce negative consequences.^29^ The impact a therapist had in assisting patients in managing nausea is also unknown. For example, anecdotal evidence suggests a patient fully immerses themselves into their experience through the guide of a therapist to alleviate nausea dates to the late 1950s-1960s but has not been validated.^27,28^

### Anxiety

Three studies identified anxiety as an adverse effect.^11,16,17^ According to 1 study, anxiety was reported in 4% of participants administered high-dose psilocybin, 8% with a moderate dose, and none with a low dose.^11^ However, another study stated 26% of participants with high-dose psilocybin and 15% with low-dose psilocybin experienced an anxiety episode.^16^ All 3 studies identifying anxiety stated that, similar to nausea, anxiety resolved between 24 and 48 hours. While the severity of anxiety was not thoroughly discussed in any of the studies, the studies mentioned that anxiety was not serious. In the data set reviewed, 1 case was identified in which a patient received a pharmacologic intervention (ie, lorazepam, 2 mg) after taking high-dose psilocybin and experiencing acute anxiety.^11^ In general, the study protocols discussed using medications to treat anxiety not resolving after nonpharmacologic interventions (eg, guidance from therapist). For example, Goodwin et al^11^ noted benzodiazepine anxiolytics, such as lorazepam or alprazolam, given orally may be preferred due to rapid onset, short duration of action, and the possibility that another route (eg, intravenous injection) may exacerbate anxiety. Two additional studies listed oral diazepam, 5 to 10 mg, and oral olanzapine, 5 to 10 mg, as rescue medications for severe adverse psychological distress or severe anxiety in the protocols.^17,19^ Another study mentioned diazepam and risperidone as rescue medications for anxiety or psychosis, with no dosing range.^15^ Carhart-Harris et al^18^ listed lorazepam (oral or injectable) as a rescue medication for treating events of severe panic that would place people at risk after not responding to psychological intervention. Perhaps having the therapist present may assist in decreasing or managing anxiety through simple arm-holding for patients experiencing anxiety^18^; however, given the increase in anxiety, there may be a need to provide such pharmacologic transparency on protocols designed to manage anxiety not controlled by the therapist.

### Dizziness

Although a recent meta-analysis focused on dose-dependent response did not report dizziness as an adverse effect of psilocybin,^8^ our analysis found it statistically significant. Two studies identified dizziness as an adverse effect.^11,19^ One study reported 6% dizziness with high-dose psilocybin, 1% with moderate dose and none with low dose.^11^ The other study reported 8% of participants with dizziness after administration of psilocybin.^19^ Both studies stated dizziness resolved between 24 and 48 hours. Neither study used any medication to treat dizziness, and both reported it was a nonserious adverse effect. None of the study protocols defined any medications to treat severe dizziness. Similar to their interventions with other adverse effects, the role a therapist has in managing dizziness (eg, asking patients to lie down, encouraging them to trust the experience) is also unknown. Some protocols state that a patient should lie down with eye coverings,^11,16,17,19^ and therefore such measures may be enough to decrease the severity of the dizziness and avoid requiring any medications.

### Additional Adverse Effects

Two studies reported elevated blood pressure and heart rate.^16,17^ In one trial, 76% of participants experienced elevated blood pressure at a therapeutic dose of 21 mg.^17^ In another study, 34% of participants had elevated systolic blood pressure (>160 mm Hg) and 13% had elevated diastolic blood pressure (>100 mm Hg) with high-dose psilocybin.^16^ However, patients taking low-dose psilocybin (in control group) also had elevated systolic (17%) and diastolic (2%) blood pressure. There is a possibility the blood pressure increases in the low-dose psilocybin (part of control group) in one study^16^ but absent in the other^17^ (using niacin as the control) may have contributed to the high heterogeneity. The observed high *I*^2^ statistic points to heterogeneity among the studies because only 2 studies contributed to this result. The limited number of studies and difference in control groups may inflate the heterogeneity measure; therefore, the findings should be interpreted within the context of this limitation. However, recent findings suggest an increased blood pressure dose-related response with a relative risk of 1.04% (*P* = .04) appear to support our results.^8^ Peak heart rate was 71 beats/min at 300 minutes postdosing in one study, while peak heart rate was 84 beats/min postdosing using high-dose psilocybin in another.^16,17^ Elevated heart rate in both studies was not considered serious and resolved within 24 hours. Additionally, 1 of the studies noted elevated blood pressure with the moderate dose, which was reported as nonsignificantly different from placebo.^19^ However, the sample size of this adverse effect was not described in the study or supplemental materials and therefore not included in the meta-analysis. Also, 4 of the 6 studies^11,15,16,17^ excluded patients with uncontrolled hypertension or elevated blood pressure at baseline and, therefore, the effects of psilocybin on blood pressure need to be further explored. At this time, we are not aware of any medications used to treat this effect, even though elevated heart rate has been described in the literature.^17^ Despite limited data on listing medications to treat increased blood pressure or heart rate, based on its mechanism of action and pharmacokinetic profile, clonidine may be an option for psychedelic-induced increased blood pressure and/or heart rate but has only been studied in mice.^30^ Protocols and guidelines note oral nifedipine, 10 mg, or intravenous labetalol as rescue medications for hypertension.^19,23^ Data suggest both clonidine and nifedipine are equally effective to treat urgent hypertension in general.^31^ While dosing per se has not been studied in this target population, based on studies and the pharmacokinetic profile of psilocybin, clonidine, 0.1 to 0.2 mg, orally or nifedipine, 10 to 20 mg, orally per dose appears reasonable and recommended for short-term resolution.^31,32^

Other adverse effects analyzed in the meta-analysis but not showing a significant difference included paranoia and transient thought disorder. Three studies reported a total of 3 cases of paranoia with high-dose psilocybin across 128 patients.^15,16,17^ Additionally, 5 patients in 2 studies totaling 103 patients experienced transient thought disorder with psilocybin (1 with high dose, 4 with moderate dose).^11,17^ One study listed risperidone, while another reported olanzapine and diazepam as agents to treat psychological distress.^11,17^ Risperidone can also be a potential option in treating acute psychological distress.^11^ While the incidence of both paranoia and transient thought disorder appears to be low, this may be an adverse effect worth monitoring in the future and supported by a recent study suggesting a dose-response relationship.^8^ All 6 studies used a therapist/facilitator to assist patients during treatment. The use of these therapists may have played a role in supporting these cases and preventing increased severity or complications. Published guidelines provide recommendations for study personnel to assist participants during their psychedelic experience.^23^ Additionally, there are a number of certificate programs designed to provide psychedelic psychotherapy training.^33^ None of the studies stated whether any of the therapists had any specific psychedelic certifications. Therefore, the utility of such certifications also merits further study in managing patient response to adverse effects.

Single studies reported other acute adverse effects, including visual perceptive effects (44%), physical discomfort (21%), fatigue (approximately 6%), euphoric mood (approximately 5%), and mood alteration (approximately 5%).^11,15,16^ While these adverse effects were only identified in single studies, it is unknown whether they were specifically evaluated across other studies, and it is difficult to speculate any dose-related response. Regardless, all the adverse effects identified in single trials were not considered serious and resolved within 24 hours with no pharmacologic treatment needed. Visual perceptual effects, an expected adverse effect of psilocybin, was only identified in 1 study.^15^ While the incidence occurred in 44% of the participants, 6% continued to experience symptoms after the dosing day and resolved by day 9.

### Strengths and Limitations

Overall, the strengths of this study include the ability to evaluate the adverse event profile of therapeutic doses of psilocybin using a meta-analysis approach when possible given the currently small number of studies with limited sample sizes. However, these studies had a low risk of bias. There are several limitations to our study results. First, our meta-analysis is based on 6 randomized controlled studies published only in English, which have less sample sizes for analysis to conclude the potential adverse effects caused by psilocybin. Additionally, the studies focus more on acute adverse effects, usually concentrating on the first 48 hours and appear to be less stringent with time. There are some adverse effects that are mentioned in only 1 study and cannot be further analyzed. A bias within the studies for focusing on certain adverse effects and not others may be possible. Selection bias may also be a limitation. Participants in these studies have been predominantly White adults without comorbidities that may be exacerbated (eg, hypertension) with psilocybin use. Also, since psilocybin appears to act as an antidepressant, future studies need to evaluate suicidality that, although rare, may pose a risk in younger adults and is a boxed warning on all antidepressants. This is of particular interest since 5 of the 6 studies appeared to exclude patients with potential suicide risk.^11,15,16,18,19^ Additionally, there is a lack of recent research data discussing the treatment of psychedelic adverse effects. One study describes safety guidelines for hallucinogen research, and while rescue medications are briefly mentioned, the guidelines primarily focus on participant selection and preparation, study personnel and appropriate conduct, physical environment, and postsession procedures. It is also unknown whether future studies exploring higher doses or more frequent use of psilocybin may carry additional adverse effects or increase the severity of symptoms. Furthermore, it is important to assess the impact a therapist may have in mitigating any of these adverse effects.

## Conclusions

In this systematic review and meta-analysis, therapeutic doses of psilocybin appeared to produce tolerable acute adverse effects that typically resolved within 24 to 48 hours. However, less common adverse effects, such as paranoia and prolonged visual perceptual effects, warrant attention. Larger trials are necessary to fully assess these adverse effects, particularly in populations with comorbid health conditions. Recommendations for solicited acute adverse effects should, at a minimum, include headache, nausea, anxiety, dizziness, paranoia, blood pressure and/or heart rate changes, visual perceptual effects, physical discomfort, and mood changes. Although infrequent, the possibility of suicidality, prolonged paranoia, and persistent visual perceptual effects should be monitored over the long term. The effectiveness of medications and alternative treatments in managing these symptoms requires further investigation. Additionally, the role of licensed therapists in managing adverse effects presents an avenue for future research.

## Notes

Supplement 1. **eFigure 1.** Sensitivity Analysis **eFigure 2.** Funnel Plots

Supplement 2.
Data Sharing Statement
