# Supplementary material for: Acute Adverse Effects of Therapeutic Doses of Psilocybin: A Systematic Review and Meta-Analysis
Source: JAMA Netw Open. 2024 Apr 10;7(4):e245960. doi: 10.1001/jamanetworkopen.2024.5960 (PMC11007582; doi:10.1001/jamanetworkopen.2024.5960)
Supplement: Supplement 1. — eFigure 1. Sensitivity Analysis eFigure 2. Funnel Plots [file jamanetwopen-e245960-s001.pdf]

## Supplementary Online Content

Yerubandi A, Thomas JE, Bhuiya NMMA, Harrington C, Zapata LAV, Caballero J. Acute adverse effects of therapeutic doses of psilocybin: a systematic review and meta-analysis. *JAMA Netw Open*. 2024;7(4):e245960.
doi:10.1001/jamanetworkopen.2024.5960

**eFigure 1.** Sensitivity Analysis

**eFigure 2.** Funnel Plots

This supplementary material has been provided by the authors to give readers additional information about their work.

© 2024 Yerubandi A et al. *JAMA Network Open.*

**eFigure 1. Sensitivity analysis**

Headache

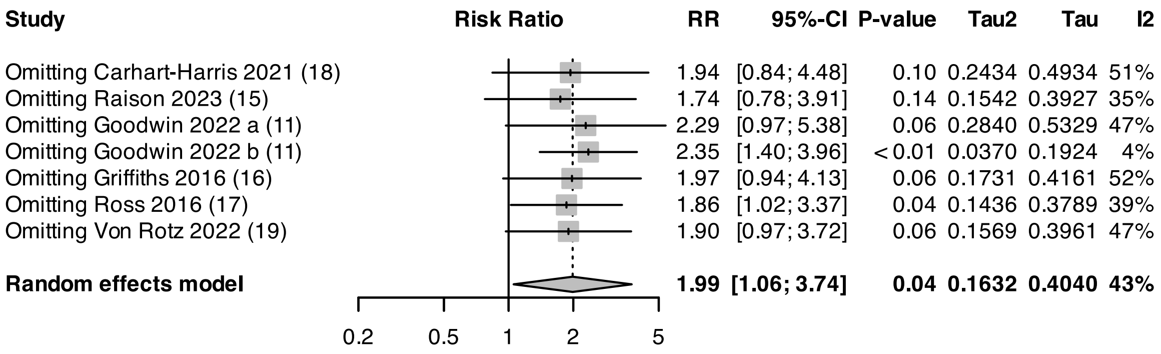


Nausea

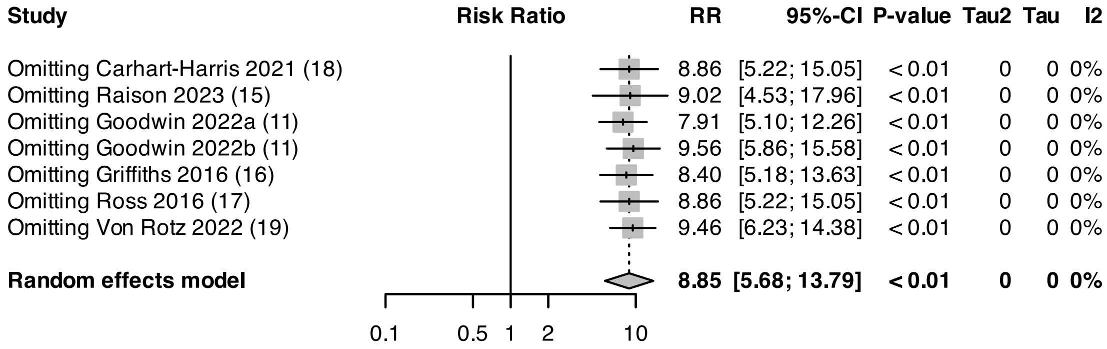


Anxiety

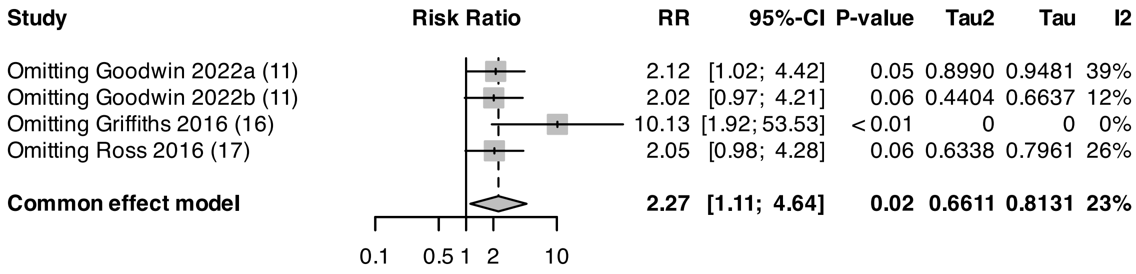


© 2024 Yerubandi A et al. *JAMA Network Open.*

Dizziness

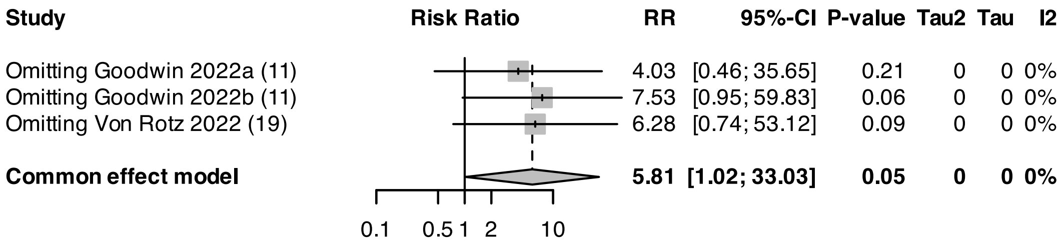


Blood Pressure

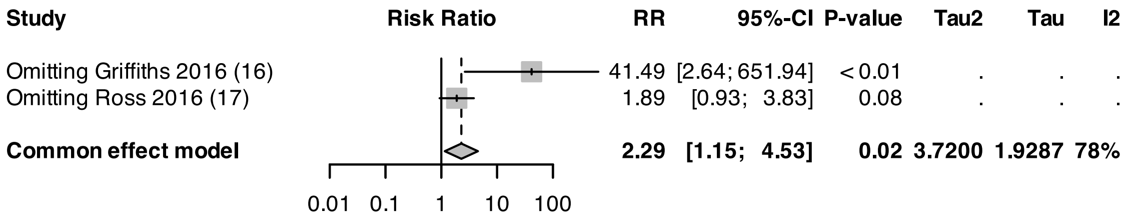


Paranoia

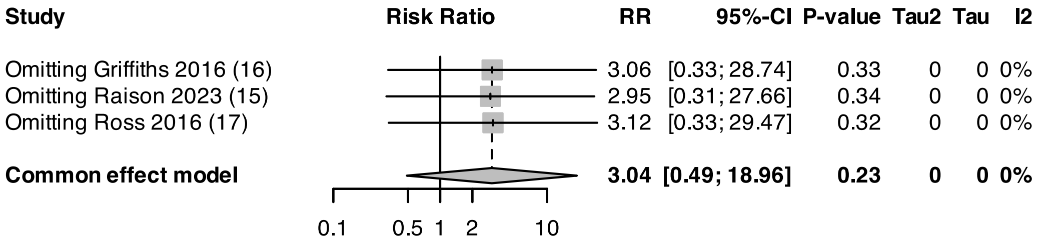


© 2024 Yerubandi A et al. *JAMA Network Open.*

Transient Psychotic Disorder

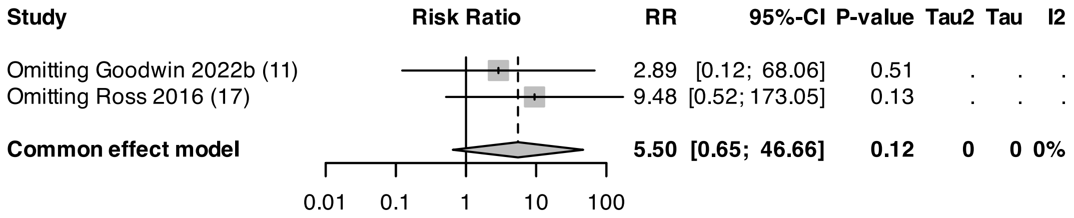


Note: Goodwin 2022a= high-dose psilocybin; Goodwin 2022b=moderate-dose psilocybin

**eFigure 2. Funnel plots**

Headache

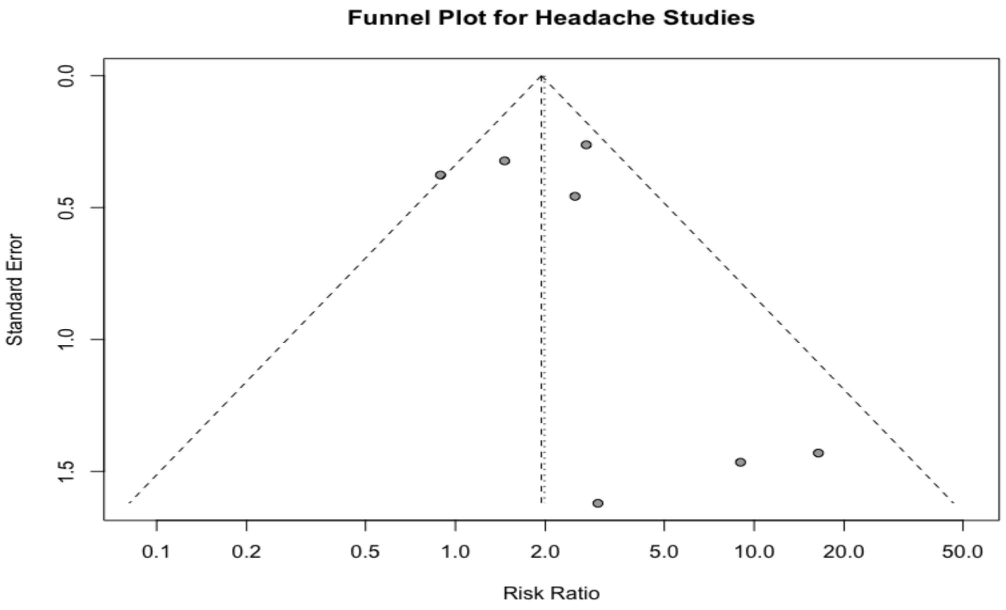


Nausea

© 2024 Yerubandi A et al. *JAMA Network Open.*

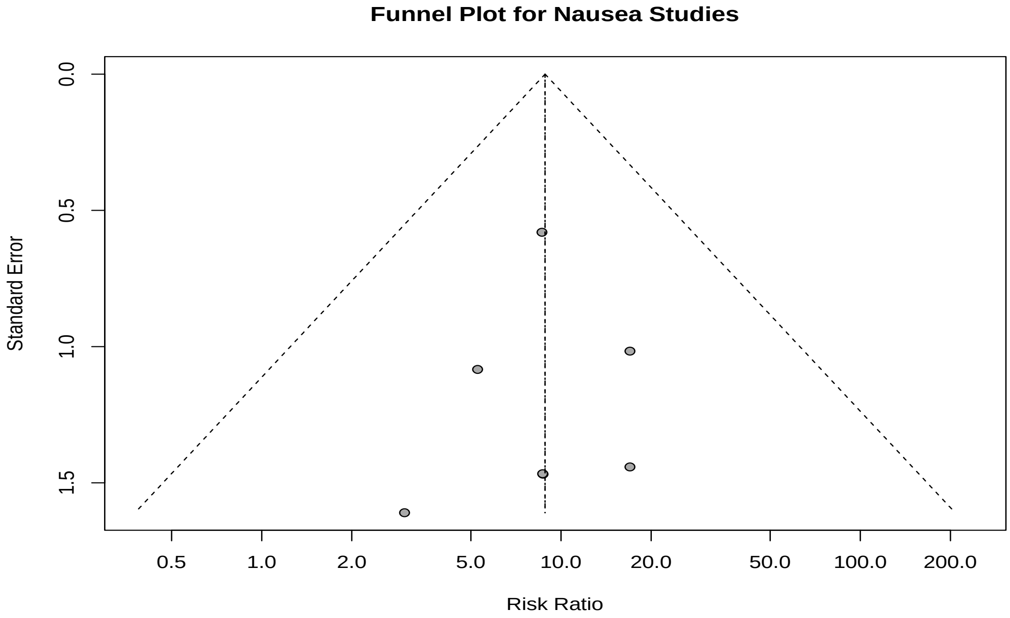


Anxiety

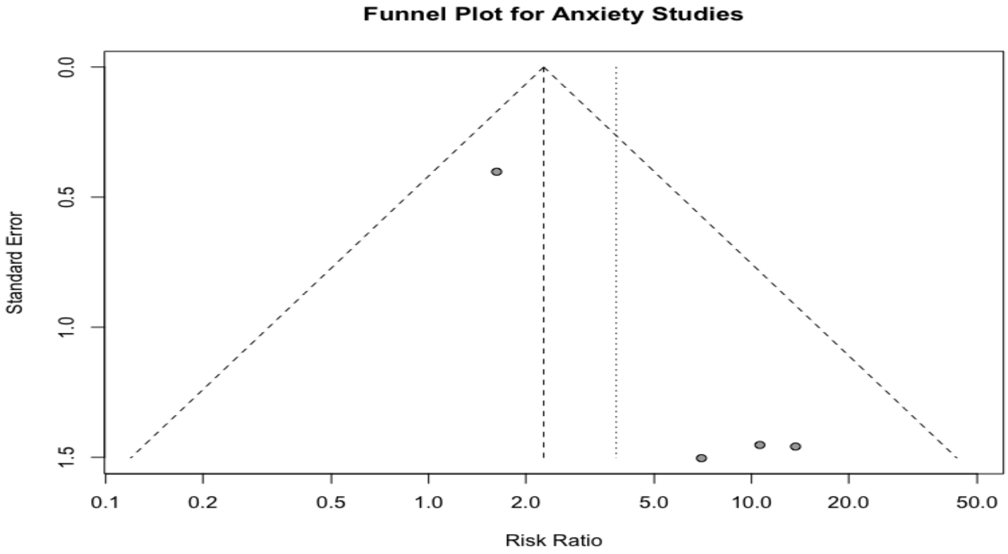


Dizziness

© 2024 Yerubandi A et al. *JAMA Network Open.*

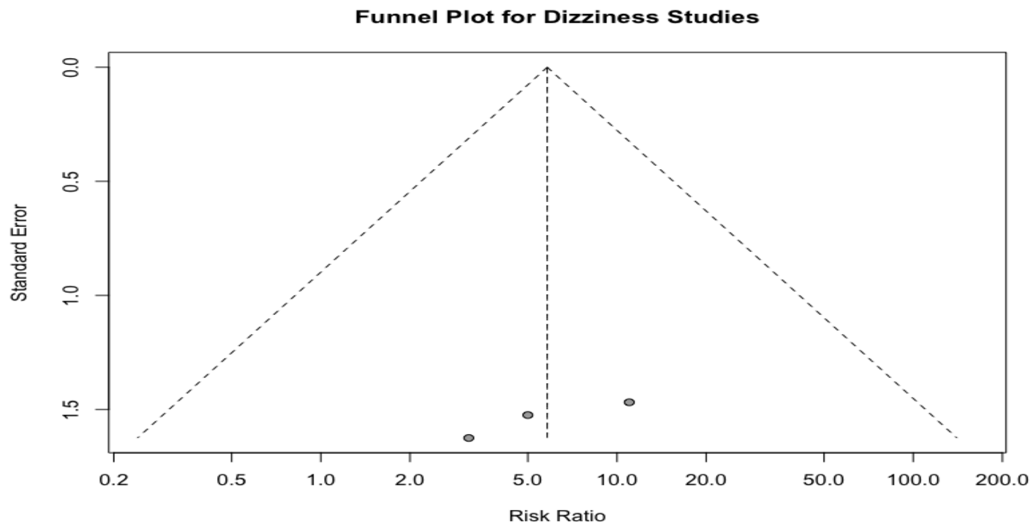


Blood Pressure

© 2024 Yerubandi A et al. *JAMA Network Open.*

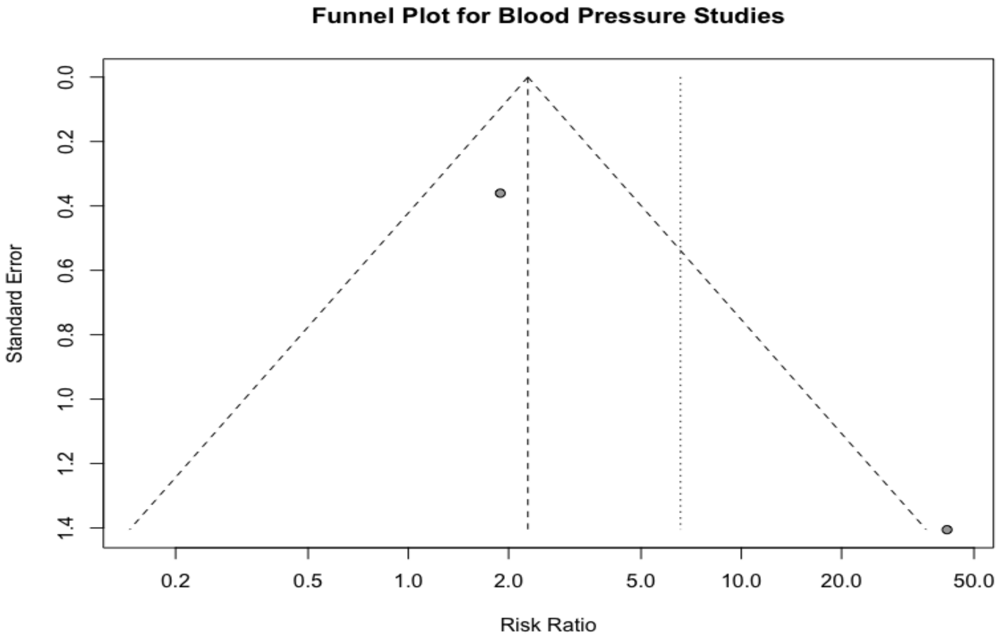


Paranoia

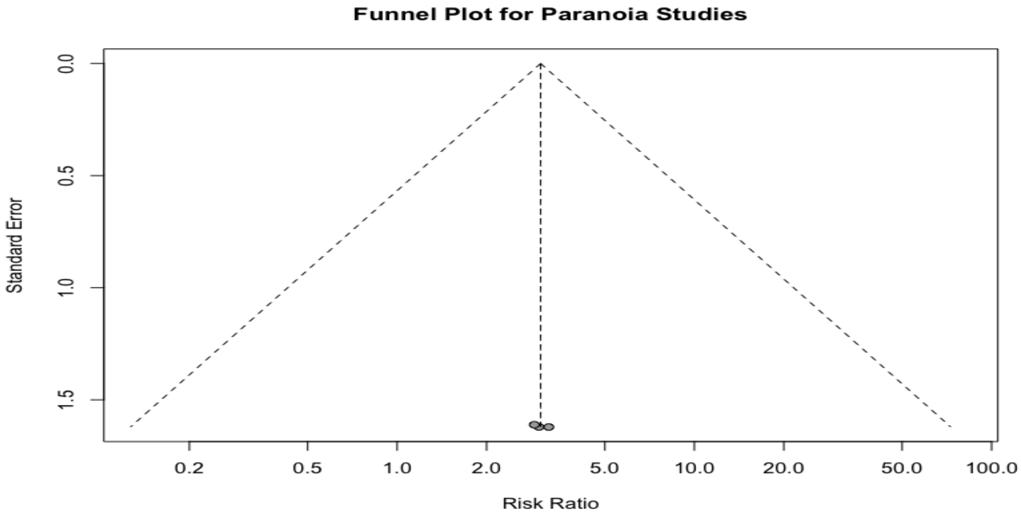


Transient Thought Disorders

© 2024 Yerubandi A et al. *JAMA Network Open.*

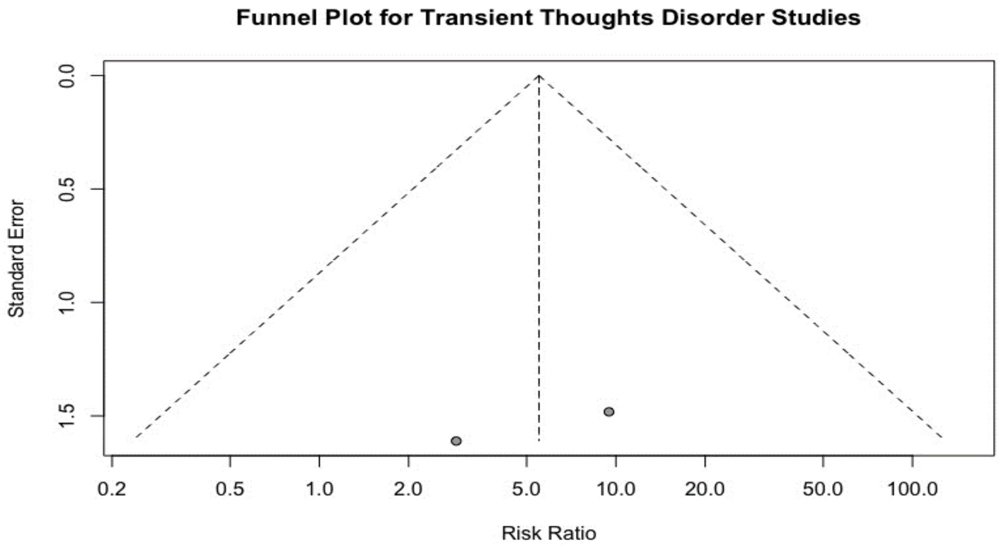


© 2024 Yerubandi A et al. *JAMA Network Open.*
