# Supplementary material for: Acute Adverse Effects of Therapeutic Doses of Psilocybin: A Systematic Review and Meta-Analysis
Source: JAMA Netw Open. 2024 Apr 10;7(4):e245960. doi: 10.1001/jamanetworkopen.2024.5960 (PMC11007582; doi:10.1001/jamanetworkopen.2024.5960)
Supplement: Supplement 2. — Data Sharing Statement [file jamanetwopen-e245960-s002.pdf]

Data Sharing Statement

Yerubandi. Acute Adverse Effects of Therapeutic Doses of Psilocybin. *JAMA Netw Open*. Published April 10, 2024. doi:10.1001/jamanetworkopen.2024.5960

Data
Data available: Yes
Data types: Other (please specify)
Additional Information: It is a meta-analysis. Data are easily available.
How to access data: It is a meta-analysis. Data are easily available.
When available: With publication

Supporting Documents
Document types: None

Additional Information
Who can access the data: It is a meta-analysis. Data are easily available online.
Types of analyses: It is a Meta-Analysis. Data are presented in the manuscript.
Mechanisms of data availability: N/A
Any additional restrictions: N/A
